# A comparison of liquid and solid culture for determining relapse and durable cure in phase III TB trials for new regimens

**DOI:** 10.1186/s12916-017-0955-9

**Published:** 2017-11-24

**Authors:** Patrick P. J. Phillips, Carl M. Mendel, Andrew J. Nunn, Timothy D. McHugh, Angela M. Crook, Robert Hunt, Anna Bateson, Stephen H. Gillespie

**Affiliations:** 1 MRC Clinical Trials Unit at UCL, London, UK; 2 Division of Pulmonary & Critical Care Medicine, University of California, San Francisco, San Francisco, USA; 3 Global Alliance for TB Drug Development, New York, NY USA; 4 Centre for Clinical Microbiology, UCL, London, UK; 5 School of Medicine, University of St Andrews, St Andrews, UK; 6 Division of Biostatistics, University of California, San Francisco, San Francisco, USA

**Keywords:** Tuberculosis, MGIT, LJ, Clinical trials, Relapse, Culture media

## Abstract

**Background:**

Tuberculosis kills more people than any other infectious disease, and new regimens are essential. The primary endpoint for confirmatory phase III trials for new regimens is a composite outcome that includes bacteriological treatment failure and relapse. Culture methodology is critical to the primary trial outcome. Patients in clinical trials can have positive cultures after treatment ends that may not necessarily indicate relapse, which was ascribed previously to laboratory cross-contamination or breakdown of old lesions. Löwenstein-Jensen (LJ) medium was the previous standard in clinical trials, but almost all current and future trials will use the Mycobacteria Growth Indicator Tube (MGIT) system due to its simplicity and consistency of use, which will affect phase III trial results.

LJ was used for the definition of the primary endpoint in the REMoxTB trial, but every culture was also inoculated in parallel into the MGIT system. The data from this trial, therefore, provide a unique opportunity to investigate and compare the incidence of false ‘isolated positives’ in liquid and solid media and their potential impact on the primary efficacy results.

**Methods:**

All post-treatment positive cultures were reviewed in the REMoxTB clinical trial. Logistic regression models were used to model the incidence of isolated positive cultures on MGIT and LJ.

**Results:**

A total of 12,209 sputum samples were available from 1652 patients; cultures were more often positive on MGIT than LJ. In 1322 patients with a favourable trial outcome, 126 (9.5%) had cultures that were positive in MGIT compared to 34 (2.6%) patients with positive cultures on LJ. Among patients with a favourable outcome, the incidence of isolated positives on MGIT differed by study laboratory (*p* < 0.0001) with 21.9% of these coming from one laboratory investigating only 4.9% of patients. No other baseline factors predicted isolated positives on MGIT after adjusting for laboratory. There was evidence of clustering of isolated positive cultures in some patients even after adjusting for laboratory, *p* < 0.0001. The incidence of isolated positives on MGIT did not differ by treatment arm (*p* = 0.845, unadjusted). Compared to negative MGIT cultures, positive MGIT cultures were more likely to be associated with higher grade TB symptoms reported within 7 days either side of sputum collection in patients with an unfavourable primary outcome (*p* < 0.0001) but not in patients with a favourable outcome (*p* = 0.481).

**Conclusions:**

Laboratory cross-contamination was a likely cause of isolated positive MGIT cultures which were clustered in some laboratories. Certain patients had repeated positive MGIT cultures that did not meet the definition of a relapse. This pattern was too common to be explained by cross-contamination only, suggesting that host factors were also responsible. We conclude that MGIT can replace LJ in phase III TB trials, but there are implications for the definition of the primary outcome and patient management in trials in such settings. Most importantly, the methodologies differ in the incidence of isolated positives and in their capacity for capturing non-tuberculosis mycobacteria. It emphasises the importance of effective medical monitoring after treatment ends and consideration of clinical signs and symptoms for determining treatment failure and relapse.

**Electronic supplementary material:**

The online version of this article (doi:10.1186/s12916-017-0955-9) contains supplementary material, which is available to authorized users.

## Background

Tuberculosis kills more people than any other infectious disease worldwide [1]. Identifying new shorter and safer treatment regimens is essential for making progress in controlling the disease. New regimens are being developed and need to be evaluated in phase III clinical trials. It is accepted in regulatory guidance and in recent phase III trials [2–5] that the primary efficacy endpoint is a composite outcome that includes mainly bacteriological treatment failure and relapse. Choice of bacteriological culture method is, therefore, critical for the primary endpoint of phase III trials. Previously this endpoint was defined by culture usually on Löwenstein-Jensen (LJ) medium [6]; the quality and sensitivity of this medium varies significantly depending on its source [7].

It is recognised that patients intensively studied in clinical trials can have positive cultures in the follow-up period that may not necessarily indicate clinical relapse [8, 9]. Typically, subsequent cultures are negative, there is no evidence of symptomatic recurrence and these patients do not need to be retreated. Such post-treatment positives have been attributed to laboratory cross-contamination leading to a false positive or to the breakdown of an old cavity, releasing organisms into the sputum from a patient who has no signs and symptoms of TB and will eventually be classified as having a favourable outcome, therefore defined as an isolated positive. Evidence for both of these explanations has been obtained recently using whole genome sequencing of recurrence strains in a clinical trial [10]. Based on this knowledge, bacteriological relapse in a phase III clinical trial continues to be defined as two positive cultures on solid media at separate visits without an intervening negative culture [2–5], in line with definitions from earlier trials [6].

In the REMoxTB phase III randomised controlled trial [4], LJ was used for the definition of the primary endpoint for continuity with previous trials, but the Mycobacteria Growth Indicator Tube (MGIT) system was used in parallel to assess its utility for future trials in view of its simplicity of use and consistent formulation and quality control. We have previously shown in this trial that differences between regimens and therefore the primary efficacy results were the same irrespective of the detection method [4]. However, to investigate more specifically the comparative incidence of isolated positives in liquid and solid media, and their potential impact on the primary efficacy results, we reviewed all post-treatment positive cultures to understand more fully the impact of using MGIT, which is increasingly used in trials as the medium of choice [11, 12].

## Methods

The REMoxTB trial (Clinicaltrials.gov NCT00864383) was a randomised placebo-controlled double-blind trial to test whether two 4-month regimens substituting moxifloxacin for either ethambutol or isoniazid were non-inferior to the standard 6-month four-drug regimen, as described previously [4, 13]. A total of 1931 patients were randomised across sites in Africa and Asia and followed for 18 months from randomisation. The trial showed that the two 4 month moxifloxacin regimens were safe but did not have non-inferior efficacy compared to the 6 month control in patients with uncomplicated, smear-positive tuberculosis [4].

During the trial, sputum samples were taken for smear and culture (LJ and MGIT in parallel) weekly to 8 weeks during treatment, monthly thereafter to 6 months and 3-monthly thereafter to 18 months from randomisation. One sputum sample was collected and inoculated into both LJ and MGIT. Sputum induction was not performed. The clinical and laboratory methodology has been described previously [4], with laboratory procedures described in full in the REMoxTB laboratory manual (https://www.ucl.ac.uk/infection-immunity/research/res_ccm/ccm_accor/ccm_remox, accessed 12 June 2017). The per protocol definition of the primary outcome of favourable and unfavourable was used in the current investigation, as this was closest to a purely bacteriological outcome. Any one of the following was classified as an unfavourable outcome: culture-confirmed or clinical treatment failure; culture-confirmed relapse; death from TB or respiratory distress during post-treatment follow-up; non-violent death during treatment; retreatment for TB with or with culture confirmation. A favourable outcome was defined as a patient having at least two negative culture results at different visits without an intervening positive culture at the end of follow-up, not having otherwise had an unfavourable outcome.

For the current investigation, post-treatment follow-up cultures were defined as all culture results at or after 33 weeks from randomisation, since the first post-treatment follow-up visit that included all patients in the trial was at 39 weeks (month 9), and this visit could occur as early at 33 weeks, accounting for the visit window. Positive cultures from patients with an unfavourable outcome (and therefore indicative of relapse and/or treatment failure) were considered separately from those from patients with a favourable outcome. The latter were considered isolated positive cultures, since the patients had subsequent negative cultures and were ultimately classified as having been cured at the end of follow-up without the need for additional treatment.

For the primary efficacy endpoint, favourable and unfavourable outcomes were defined using LJ medium. For this reason, contaminated, missing or suspect values for LJ results were monitored and patients were often brought back for repeat sampling. This occurred particularly at the end of follow-up to ensure that patients who were doing well had documented negative cultures on LJ to meet the definition of a favourable outcome on the primary endpoint. This was not done for MGIT results, since they were not used for the primary endpoint. In addition, a decision to restart treatment (which would meet the definition of an unfavourable outcome) was most often based on the LJ results and clinical considerations without regard to the MGIT results.

### Statistical methods

Logistic regression was used to model incidence of post-treatment positive cultures including, where appropriate, a patient-level random intercept. The likelihood ratio test was used to compare models. The following baseline covariates were evaluated as predictors of positive cultures: treatment arm, sex, presence of cavitation, history of smoking, current smoking status, race, HIV status, weight, age, CD4 count, BMI, weight band, baseline MGIT days to positivity. The likelihood ratio test was used to compare models and forward and backwards stepwise selection used to develop the model that best fit the data. The χ^2^ test for independence was used to evaluate the association between the result of MGIT cultures and the paired smear or LJ result at the same visit or the highest grade of TB symptoms reported within 7 days of sputum collection. TB symptoms included any of the following seven: cough, haemoptysis, fever, night sweats, shortness of breath, chest pains, unintentional weight loss.

## Results

Considering paired results from the same sputum samples in post-treatment follow-up visits, cultures were more often positive in MGIT culture than on LJ. Of 12,209 sputum samples across all 1652 patients, 638 (5.2%) were positive in both media, while 305 (2.5%) were positive in MGIT but negative on LJ, and only 29 (0.2%) were positive on LJ and negative in MGIT; 152 (1.2%) were contaminated in both media, while 1196 (9.8%) were contaminated on LJ and positive or negative in MGIT, and 570 (4.7%) were contaminated in MGIT and positive or negative on LJ. An additional 624 (5.1%) of samples were MGIT false positive (MGIT instrument positive, but no organisms detected; these were classified in the results the same as contaminated) and positive or negative on LJ. Non-TB mycobacteria (NTM) were more often identified in MGIT than on LJ, 318 (2.6%) and 88 (0.7%) respectively in all samples, but only in 35 (0.3%) in both samples. Excluding those samples where one result was contaminated or missing, there was agreement between LJ and MGIT in 8801 (93.5%) of 9404 samples.

Of 1322 patients with a protocol-defined favourable outcome on LJ, 126 (9.5%) had post-treatment follow-up samples that were positive in MGIT, compared with 34 (2.6%) who had positive samples on LJ (Table 1). Twenty-four patients (1.8%) had two or more positive MGIT cultures in post-treatment follow-up on different visits (Table 1) compared to 1 (<0.1%) with two or more positive LJ cultures. The two positive LJ cultures were separated by a negative LJ culture, and therefore this did not constitute an unfavourable outcome. Of the 24 patients with multiple positive cultures in MGIT, 9 had intervening negative MGIT cultures between the positive MGIT cultures, 11 had two positive MGIT cultures without an intervening negative and 4 had more than two positive MGIT cultures in a row without intervening negative cultures — despite all these patients having a favourable outcome with no need for retreatment.

**Table 1:** Number of patients with positive cultures at or after week 33 (lower bound of the month 9 visit window) on separate visits in patients with a favourable outcome (per protocol) by culture medium. Two positive cultures on the same day only count as a single result

Culture medium	Number of positive cultures at or after week 33 on culture medium in patients with a favourable outcome					
	0	1	2	3	4	Total
MGIT	1196 (90%)	102 (8%)	17 (1%)	5 (<0.5%)	2 (<0.5%)	1322
LJ	1288 (97%)	33 (2%)	1 (<0.5%)	0	0	1322

**Fig. 1:**
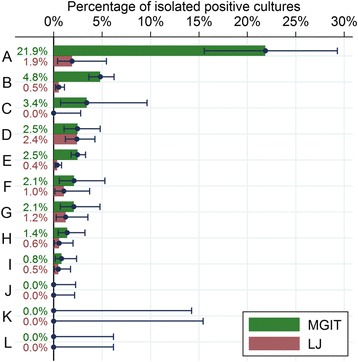
Percentage of isolated positive cultures by culture media and study laboratory, defined as the percentage of cultures at or after week 33 (excluding those with contaminated or missing results) that were positive among patients classified as favourable on the per protocol primary outcome. Laboratories are sorted by percentage of isolated positives on MGIT and labelled A to L. Error bars show 95% exact binomial confidence intervals

Among patients who had protocol-defined favourable outcomes, there was clear evidence that the incidence of isolated positives on MGIT differed by study laboratory (*p* < 0.0001) where in one laboratory, 21.9% of all post-treatment follow-up MGIT cultures in patients who were deemed to have favourable outcomes were positive (Fig. 1). This laboratory (A in Fig. 1) was responsible for the cultures of only 4.6% of all patients in the trial, yet it reported 8 (33%) of the 24 patients with a protocol-defined favourable outcome with two or more positive MGIT cultures in post-treatment follow-up. This indicates that laboratory cross-contamination was likely to have been a common cause of isolated positives. Furthermore, while we have previously shown that there was no evidence for an interaction between treatment and study centre in the primary outcome analysis [4], patients served by laboratory A (fewer than 100) had a 2.67 increased odds of an unfavourable outcome (95% confidence interval, CI (1.42, 5.01), *p* = 0.002, per protocol analysis, adjusted for treatment arm) as compared to other patients in the trial. This suggests laboratory cross-contamination may also have contributed to the higher number of patients being classified as bacteriological relapses. No other factors predicted isolated positives on MGIT in the model adjusted for study laboratory (all baseline covariates evaluated). There was clear evidence of clustering of isolated positive in some patients even after adjusting for study laboratory (random effects variance 1.23 (standard error 0.373), *p* < 0.0001), meaning that the number of patients with two or more positive MGIT cultures in post-treatment follow-up was too high to be explained by cross-contamination only. The incidence of isolated positives on MGIT did not differ by treatment arm (*p* = 0.845, unadjusted).

Since two consecutive positives on LJ led to a defined unfavourable outcome, it was not possible to determine whether there was clustering of isolated positives on LJ among patients who had a favourable outcome. The incidence of isolated positives on LJ also differed by study laboratory, *p* = 0.0081, although the variability between laboratories was lower than for MGIT (Fig. 1). No other factors predicted isolated positives on LJ in the model adjusted for study laboratory (all baseline covariates evaluated). In particular, incidence of isolated positives on LJ did not differ by treatment arm (*p* = 0.451, unadjusted).

**Fig. 2:**
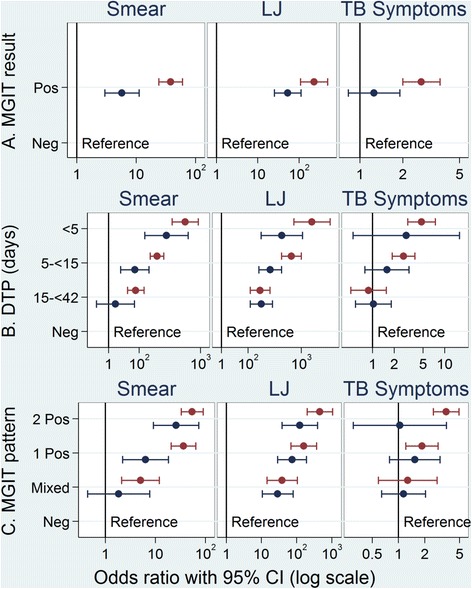
Odds ratios with 95% confidence intervals for smear positivity, LJ positivity or TB symptoms (Grade 1 or higher), separately for patients with a favourable outcome (*blue*) or unfavourable outcome (*red*) for **a** MGIT result (with negative as reference); **b** MGIT days to positivity (DTP) < 5 days, 5 to < 15 days, 15 to < 42 days with negative (42 days or more) as reference; **c** pattern of MGIT results at visit: *All negative* at least one negative result and no positive results at that visit. *1 Positive* a single positive result and no negative results at that visit, *Mixed* a single positive result and at least one negative result at that visit, *2 Positives* two positive results and no negative results at that visit

We tested the hypothesis that MGIT post-treatment positive results in patients with a favourable outcome are not indicative of relapse. Table 2 shows the analysis of the association between the result of MGIT cultures at or after week 33 and the paired LJ or smear result at the same visit, and TB symptoms reported within 7 days of sputum collection, by per protocol primary outcome. Compared to negative MGIT cultures, positive MGIT cultures were more likely to be positive on LJ or smear irrespective of per protocol primary outcome (*p* < 0.0001 in each case). However, the odds ratios of a positive smear or LJ, given a positive MGIT, were much higher in patients with an unfavourable outcome, 38.0 95% CI (24.1, 60.0) and 231.7 95% CI (109.1, 492.1) respectively, than in those with a favourable outcome, 5.8 95% CI (3.0, 11.2) and 53.4, 95% CI (25.4, 112.4) respectively (Fig. 2a). Among patients with a favourable primary outcome, however, only 7% of MGIT positive cultures at or after week 33 were smear positive when this result was available and only 13% were LJ positive. In contrast, in patients with an unfavourable primary outcome, 75% of MGIT positives were smear positive and 85% LJ positive.

**Table 2:** Association between MGIT culture results by primary outcome isolated positives on MGIT and LJ culture results, smear results and TB symptoms. Data restricted to culture results at or after week 33

Per protocol primary outcome	MGIT result	Paired smear result from same visit			Paired LJ result from same visit			Highest grade of TB symptom^a^ reported within 7 days of patient visit			
		Negative	Positive	Total	Negative	Positive	Total	Absent or < Grade 1	Grade 1 (mild)	Grades 2–4	Total
Favourable	Negative	4659 (99%)	60 (1%)	4719	4456 (100%)	12 (<0.5%)	4468	3983 (85%)	453 (10%)	225 (5%)	4661
	Positive	148 (93%)	11 (7%)	159	132 (87%)	19 (13%)	151	131 (82%)	20 (13%)	8 (5%)	159
χ^2^ test for independence				*p* < 0.0001			p < 0.0001				*p* = 0.481
Unfavourable	Negative	336 (93%)	26 (7%)	362	331 (98%)	8 (2%)	339	240 (69%)	69 (20%)	40 (11%)	349
	Positive	105 (25%)	309 (75%)	414	60 (15%)	336 (85%)	396	181 (45%)	116 (29%)	106 (26%)	403
χ^2^ test for independence				*p* < 0.0001			*p* < 0.0001				*p* < 0.0001

^a^TB symptom as reported on REMoxTB CRF including any one of the following seven: cough, haemoptysis, fever, night sweats, shortness of breath, chest pains, unintentional weight loss

Table 2 also shows that, compared to negative MGIT cultures, those with a positive MGIT result were more likely to be associated with higher grade TB symptoms reported within 7 days of sputum collection in patients with an unfavourable primary outcome (*p* < 0.0001) but, importantly, this pattern was not found in patients with a favourable outcome, where isolated positive cultures were not associated with TB symptoms (*p* = 0.481).

In order to further explore which of the parameters to which clinicians had access in the clinic might be associated with MGIT isolated positives, we conducted analyses restricted only to positive culture results at or after week 33. There is strong evidence that a MGIT positive culture in follow-up is more likely to be a true positive (associated with an unfavourable outcome) if the paired smear or LJ result is positive, if higher grade TB symptoms are reported, if the number of days to positivity (DTP) is lower or if there are two positive MGIT results at the same visit (*p* < 0.0001 in each case, Table 3). However, Table 3 also shows that none of these factors can be used to definitively identify an isolated positive (associated with a favourable outcome). Only 58% of MGIT positive results that are also smear negative are isolated positives, and only 69% of MGIT positive results that are also LJ negative are isolated positives. Similarly, isolated positives occurred in only 42% of MGIT positives where TB symptoms were absent or < Grade 1 and only 57% of MGIT positive results with DTP of greater than 15.

**Table 3:** Association among patients with isolated post-treatment MGIT positive results between primary outcome and paired information smear results, LJ culture result, TB symptoms, days to positivity on MGIT and pattern of MGIT culture results. Data restricted to culture results at or after week 33

		Per protocol primary outcome			
Paired information *N* (%)		Favourable	Unfavourable	Total	χ^2^ test for independence
Paired smear result from same visit	Negative	148 (58%)	105 (42%)	253	
	Positive	11 (3%)	309 (97%)	320	*p* < 0.0001
Paired LJ result from same visit	Negative	132 (69%)	60 (31%)	192	
	Positive	19 (5%)	336 (95%)	355	*p* < 0.0001
Highest grade of TB symptom^b^ reported within 7 days of patient visit	Absent or < Grade 1	131 (42%)	181 (58%)	312	
	Grade 1 (mild)	20 (15%)	116 (85%)	136	
	Grades 2–4	8 (7%)	106 (93%)	114	*p* < 0.0001
Days to positivity on MGIT	<5	6 (5%)	127 (95%)	133	
	5 to < 15	47 (19%)	202 (81%)	249	
	15 to < 42	94 (57%)	72 (43%)	166	*p* < 0.0001
Pattern of MGIT results at visit^a^	*Mixed*	86 (73%)	32 (27%)	118	
	*1 positive*	53 (30%)	122 (70%)	175	
	*≥2 positives*	20 (7%)	260 (93%)	280	*p* < 0.0001

^a^Patterns are as follows: *All negative* at least one negative result and no positive results at that visit, *1 positive* a single positive result and no negative results at that visit, *Mixed* a single positive result and at least one negative result at that visit, *≥ 2 positives* at least two positive results and no negative results at that visit

^b^TB symptom as reported on REMoxTB CRF any one of the following seven: cough, haemoptysis, fever, night sweats, shortness of breath, chest pains, unintentional weight loss

## Discussion

The development of urgently needed new regimens for TB is expensive and time-consuming and must be carried out against the backdrop of declining global funding for TB research and development [14]. It is therefore essential that pivotal phase III trials make efficient use of resources and yield reliable results. Critical to this is the definition of the primary endpoint making the best use of bacteriological results to distinguish between patients who are cured and patients who fail treatment or relapse. Inconsistent results due to even modest differences in laboratory methodology and processes can make trial results hard to interpret [15]. For this reason, we implemented standardised training and laboratory methodologies in the REMoxTB trial, and culture from every sputum sample in the trial was done on both LJ solid and MGIT liquid media. This has allowed us to provide a bridge between previous trials using solid LJ media and future trials which are expected to use liquid MGIT media [11, 12, 16].

As expected, there were slightly more positive cultures and more isolated positive cultures in MGIT than on LJ, although there was agreement between LJ and MGIT in the majority of sputum samples where contamination did not occur in either medium. This result is not surprising given the known performance characteristics of the MGIT system, which is associated with a lower limit of detection as well as a more rapid time to positivity [17–19]. There were also more isolated positive cultures in MGIT on multiple occasions among patients with a favourable outcome. This shows for the first time that even two or more positive results in MGIT may not be indicative of relapse.

We showed that laboratory cross-contamination was a cause of isolated positives on MGIT with clustering within some study laboratories, but we also found evidence of clustering of isolated positives on MGIT within individual patients showing that host factors were also responsible. It is thought that at least a proportion of isolated positives are derived from pulmonary lesions [8, 10], but we found that neither cavitation at baseline nor any other baseline characteristic was associated with an increased incidence of isolated positive on MGIT, after adjusting for site laboratory. More work is needed to identify the patient characteristics and host factors that are more likely to lead to isolated positives. We showed that post-treatment positive rates varied by site laboratory, indicating the importance of managing laboratory services closely. Although laboratory differences are confounded by geographical differences, it was notable that of the two laboratories that resulted in the most isolated positives on MGIT, one was from Asia and one was from Africa. Cross-contamination between samples has long been recognised as a challenge in mycobacterial laboratories, with the contamination rate varying widely [20]. Cross-contamination is a threat to the integrity of the results of a clinical trial and a risk to patients, since they may unnecessarily be given extended treatment that may include more toxic regimens. As with previous studies [18], we have also shown that the permissive environment for growing mycobacteria in MGIT leads to an increase in the number of non-tuberculosis mycobacteria isolated.

Positive cultures in MGIT were more likely to be smear positive or LJ positive in patients with both favourable and unfavourable outcomes, yet isolated positive cultures in MGIT in patients with a favourable outcome were not associated with TB symptoms reported within 7 days of sputum collection, while positive culture results in patients with an unfavourable outcome were strongly associated with TB symptoms. This suggests that many of these post-treatment positives are likely to be contaminants or sub-clinical findings not associated with a clinical manifestation of disease, even though more likely to be smear and LJ positive.

We showed that a paired positive smear or LJ result, higher grade TB symptoms, lower days to positivity or more MGIT positives at the visit were all strongly associated with relapse and could therefore be used, along with the presence of TB symptoms, by clinicians to give greater confidence in acting on a positive MGIT result. However, absence of these factors did not necessarily imply the MGIT positive result was an isolated positive result.

These data highlight the importance of clinical course and symptoms in interpreting positive cultures, particularly in MGIT, after the completion of treatment — in addition to paired smear results and MGIT days to positivity. This observation has implications both for patient management in general and for the primary endpoint definition for pivotal phase III trials, where no more than 8% of patients would be expected to have a true unfavourable outcome [4] and even a small number of erroneously classified relapses could result in a false interpretation of the trial.

There were a number of limitations with our study. LJ was used for the primary outcome of the REMoxTB trial, and therefore decisions to restart treatment after recurrence of disease were based primarily on LJ results and clinical course (although MGIT results were usually also available to study clinicians). While this meant that we were able to evaluate the incidence of positive cultures on MGIT that did not lead to unfavourable outcomes, we were not able to do this for LJ. Furthermore, patients with missing or contaminated LJ results at the end of follow-up were encouraged to return for follow-up visits to provide sputum for culture, and therefore there are fewer patients with missing results on LJ than on MGIT at the end of follow-up. In the trial an unfavourable outcome was not always bacteriologically confirmed on LJ, and so even the per protocol outcome might include a small number of cases of unnecessary retreatment. Finally, comparison of the strains of isolated positives on MGIT using Mycobacterial Interspersed Repetitive Units (MIRU) typing or whole genome sequencing would have provided more data to distinguish between strains that matched a patient’s baseline strain and strains that did not. However, given the large number of isolated post treatment positives in addition to bacteriological relapses, the cost of whole genome sequencing would be prohibitive. While this means that we cannot definitively distinguish isolated positives caused by laboratory cross-contamination (strains that differed) from those that originated from pulmonary tissue (strains that matched), we can nevertheless draw conclusions as to likely causality without strain typing by examining the patterns of isolated positives within and between patients.

## Conclusions

In summary, laboratory cross-contamination was a likely cause of isolated positives on MGIT with clustering within some study laboratories, but we also found evidence of clustering of isolated positives on MGIT within individual patients that was too high to be explained by cross-contamination only, showing that host factors were also responsible. We conclude that MGIT can replace LJ in phase III TB trials, but there are implications for the definition of the primary outcome and patient management in trials in such settings. Most importantly, the methodologies differ in the incidence of isolated positives and in their capacity for capturing non-tuberculosis mycobacteria. This emphasises the importance of effective medical monitoring after the end of treatment and consideration of clinical signs and symptoms in determining treatment failure and relapse.

## Additional file

Additional file 1: List of ethics committees that approved the REMoxTB trial protocol. (PDF 66 kb)

## Abbreviations

CRF: Case Report Form
LJ: Löwenstein-Jensen medium
MGIT: Mycobacteria Growth Indicator Tube
MIRU: Mycobacterial Interspersed Repetitive Units
