# Supplementary material for: A comparison of liquid and solid culture for determining relapse and durable cure in phase III TB trials for new regimens
Source: BMC Med. 2017 Nov 24;15:207. doi: 10.1186/s12916-017-0955-9 (PMC5701316; doi:10.1186/s12916-017-0955-9)
Supplement: Additional file 1: — List of ethics committees that approved the REMoxTB trial protocol. (PDF 66 kb) [file 12916_2017_955_MOESM1_ESM.pdf]

Country	Name of the IEC/IRB	Address of the IEC/IRB
China	Beijing Chest Hospital of Capital Medical University Ethics Committee	Ma Chang No.97, Tong Zhou District, Beijing, China
China	Shanghai Pulmonary Hospital Ethics Committee	Zheng Min No. 507, Yang Pu District, Shanghai, China
China	Tianjin CDC Biomedical Ethics Committee	Hua Yue No.6, He Dong District, TianjinChina
India	Biomedical Ethics Committee	580, Devli, East Sainik Farms, New Delhi 110062, India
India	Institutional Ethics Committee, Mahatma Gandhi Medical College and Hospital	Mahatma Gandhi Medical College & Hospital, RIICO Institutional Area, Sitapura, Jaipur 302022, India
Kenya	KEMRI Scientific Steering Committee KEMRI Ethical Review Committee	Kenya Medical Research Institute Mbagathi Road, Nairobi, Kenya
Malaysia	Medical Research & Ethics Committee, Ministry of Health Malaysia	Institute for Health Management, JalanRumah Sakit, Bangsar, 59000 Kuala Lumpur, Malaysia
Mexico	Comité de Investigación y Ética (División de Enseñanza, Investigación, Capacitación, Ética y Calidad) Hospital General de Occidente	Av. Zoquipan # 1050. Col. Seattle, Zapopan, Jalisco CP 45170
Mexico	National Jewish Health	1400 Jackson St. Denver Co. 80206USA
South Africa	Biomedical Research Ethics Committee	Govan Mbeki Building (Rm N40), University of KwaZulu Natal, Westville Campus, 4001,Durban,
South Africa	Medicines Controll Council	Room NG090 124,Civitas Building,Andries Street, Pretoria, South Africa
South Africa	Pharma Ethics	123 Amcor Road, Lyttleton Manor, 0157, Pretoria, South Africa
South Africa	University of Cape Town Human Research Ethics Committee	Room E52-24 Old Main Building, Groote Schuur Hospital, Observatory, 7925, Cape Town, South Africa
South Africa	Wits Human Research Ethics Committee	8 Blackwood Avenue , Parktown, 2193, Johannesburg, South Africa
Tanzania	Kilimanjaro Christian Medical College Research Ethics and Review Committee (KCMC)	KCM College Opposite Tumaini Restaurant & New Admin Building, Moshi, Tanzania
Tanzania	Mbeya Ethics and Research Committee	PO Box 419, Mbeya
Tanzania	National Institute for Medical Research	2448, Ocean Road, Dar Es Salaam, Tanzania
Thailand	Ethical Review Committee for Research in Human Subjects, Ministry of Public Health	The Office of the Secretary, Ethical Review Committee for Research in Human Subjects, Departemnt of Medical Services, 3rd Floor of the Building No.2, Ministry of Public Health, Tiwanon Road, Nonthaburi 11000, Thailand
Thailand	Ethical Review Committee of Chest Disease Institute, Department of Medical Services and Ministry of Public Health	39 Tiwanon Road, Amphur Muang, Nonthaburi 11000, Thailand
Thailand	Ethics Committees on Researches Involving Human Subjects, Rajavithi Hospital, Bangkok, Thailand	2 Rajavithi Road, Rajjathevi District, Bangkok 10400, Thailand
Thailand	The Khon Kaen University Ethics Committee for Human Research Faculty of Medicine, Khon Kaen University	17th Floor, Sumdetprasrinagarindtrabhoromrachonn ee BuildingSrinagarind Hospital 123 Mitraprap Road, Muang Khon Kaen 40002, Thailand
Zambia	University of Zambia Biomedical Research Ethics Committee	Ridgeway Campus, John Mbita Road, Lusaka, Zambia
Zambia	University of Zambia BiomedicalResearch Ethics Committee	Ridgeway Campus, John Mbita Road,Lusaka, Zambia
